# Pretreatment Modified Glasgow Prognostic Score for Predicting Prognosis and Survival in Elderly Patients with Gastric Cancer Treated with Perioperative FLOT

**DOI:** 10.3390/nu15194156

**Published:** 2023-09-26

**Authors:** Ebru Melekoglu, Ertugrul Bayram, Saban Secmeler, Burak Mete, Berksoy Sahin

**Affiliations:** 1 Department of Nutrition and Dietetics, Faculty of Health Sciences, Cukurova University, Adana 01250, Turkey; 2 Department of Medical Oncology, Faculty of Medicine, Cukurova University, Adana 01250, Turkey; ertugrulbayram84@gmail.com (E.B.); berksoys@hotmail.com (B.S.); 3 Department of Medical Oncology, Bahcelievler Medicalpark Hospital, Altinbas University, Istanbul 34180, Turkey; drsabansecmeler@hotmail.com; 4 Department of Public Health, Faculty of Medicine, Cukurova University, Adana 01250, Turkey; burakmete2008@gmail.com

**Keywords:** modified Glasgow prognostic score, gastric cancer, neoadjuvant chemotherapy, FLOT, elderly, prognosis, survival

## Abstract

The adverse effects of chemotherapy are more apparent in elderly patients and lead to worse prognosis and mortality. Identifying immunonutritional risk factors is of great importance in terms of treatment effectiveness, prognosis, and mortality in geriatric oncology. The modified Glasgow prognostic score (mGPS) is an immunonutritional index based on serum CRP and albumin levels. In this study, we aimed to investigate the role of mGPS in predicting prognosis and survival in elderly patients with gastric cancer receiving perioperative FLOT treatment. We retrospectively enrolled 71 patients aged over 65 years and grouped them according to their pretreatment mGPS score. Kaplan-Meier and Cox regression analysis showed overall survival was significantly worse in the mGPS 1 and mGPS 2 groups than in the mGPS 0 group (*p* = 0.005 and *p* < 0.001, respectively). Compared to the mGPS 0 group, the mGPS 1 group had a 6.25 times greater risk of death (95% CI: 1.61–24.28, *p* = 0.008), and the mGPS 2 group had a 6.59 times greater risk of death (95% CI: 2.08–20.85, *p* = 0.001). High BMI was identified as a significant risk factor for being in the mGPS 2 group (OR: 1.20, 95% CI: 1.018–1.425, *p* = 0.030). In conclusion, elevated pretreatment mGPS was associated with poor overall survival in elderly patients with gastric cancer treated with perioperative FLOT therapy. As such, pretreatment mGPS can be a simple and useful tool to predict mortality in this specific patient group.

## 1. Introduction

Gastric cancer ranks as the fifth most prevalent cancer and the third leading cause of cancer-related deaths globally, with an annual incidence exceeding one million cases [1]. 65% of gastric cancer cases are diagnosed at locally advanced or advanced stages and have a poor prognosis [2]. In the early stage, the main treatment is surgery, and multimodal treatments, including adjuvant, neoadjuvant chemotherapy, and radiotherapy treatments, improve survival rates. Previously, neoadjuvant chemotherapy has been shown to provide 5-year overall survival (OS) rates ranging from 36% to 38% in cases of early-stage gastric cancer [3,4]. The FLOT regimen as neoadjuvant therapy in operable gastric cancers was demonstrated by the Medical Research Council Adjuvant Gastric Infusional Chemotherapy (MAGIC) study that 5-Fluorourasil (5-FU), leucovorin, oxaliplatin, and docetaxel were effective [3], and later became the standard treatment as it showed a survival advantage over other chemotherapy regimens with the FLOT4-Arbeitsgemeinschaft Internistische Onkologie (AIO) study [5]. In these studies, the FLOT regimen has been shown to be effective and tolerable in geriatric patients with an Eastern Cooperative Oncology Group (ECOG) score of 0 or 1, which constitutes approximately 20–30% of all patient groups [3,5].

There are several predictive prognostic factors to identify high-risk gastric cancer, including age, gender, pretreatment weight, primary tumor site, tumor size, number of positive and negative lymph nodes resected, negative surgical margins, depth of invasion, and lymphovascular invasion [6,7,8]. Inflammation, an important cause of cancer development and progression, is also associated with poor prognosis [9]. Inflammatory markers such as lymphocytes, neutrophils, or inflammation-related C-reactive protein (CRP) and albumin have prognostic significance in cancer patients and have also been used as mortality indicators [10,11]. In addition, many inflammatory indices such as platelet/lymphocyte ratio (PLR), CRP/albumin ratio (CAR) [12], inflammatory prognostic index (IPI) [13], prognostic nutritional index (PNI) [14], and controlling nutritional status score (CONUT) [15] have been used as prognostic and mortality markers in gastric cancer patients. Despite all these prognostic indicators, novel markers are needed as the response to treatment and clinical course of patients differ. Recently, the modified Glasgow prognostic score (mGPS) in cancer patients has been used as a new indicator to determine the prognosis and survival of patients [16,17,18]. The prognostic value of mGPS was also confirmed in gastric cancer. However, its prognostic value in patients receiving neoadjuvant treatment is unknown [19]. However, there are no sufficient clinical studies and real-time patient experience in the literature regarding indicators that predict prognosis and survival in elderly patients with operable gastric cancer treated with the perioperative FLOT regimen. There is also a need for a prognostic tool to guide the decision-making process regarding the allocation of neoadjuvant therapy in this patient population. In this study, we aimed to investigate the importance of mGPS on prognosis and mortality in elderly patients with locally advanced gastric cancer treated with perioperative FLOT therapy.

## 2. Materials and Methods

### 2.1. Patients and Datasets

This study retrospectively enrolled 71 patients with gastric cancer aged over 65 years who were treated with perioperative FLOT at Cukurova University Balcalı Hospital from January 2013 to July 2023. We included patients over 65 years of age with locally advanced (stage 2 and 3) gastric and gastric esophageal junctional cancer who accepted preoperative chemotherapy treatment, were compatible with treatment, and had an ECOG performance score of 0 or 1. The criteria for exclusion of patients are as follows: (i) under 65 years of age, patients with (ii) early stage (stage 1) gastric cancer, (iii) metastatic disease and previous surgery, (iv) liver and kidney failure, stage 3–4 heart failure and stage 3–4 chronic obstructive pulmonary disease (COPD), (v) a low ECOG performance score (ECOG ≥ 2), (vi) missing data on the biochemical and pathological findings shown in Table 1, and (vii) patients who could not adapt to treatment (mental health problems, hypersensitivity to drugs, etc.).

The perioperative FLOT regimen required the following drugs to be administered biweekly for four cycles in both the preoperative and postoperative periods; “Docetaxel at a dose of 50 mg/m^2^ (1 h), oxaliplatin at a dose of 85 mg/m^2^ (2 h), and folinic acid at a dose of 200 mg/m^2^ (2 h) by intravenous infusion on the first day, followed by a 24-h intravenous infusion of 2600 mg/m^2^ of 5-fluorouracil”.

Biochemical, radiological, and pathological findings of the patients were collected from medical records. The Union for International Cancer Control (UICC) tumor node metastasis (TNM) classification was used for the classification of clinicopathological factors [20]. Complications after neoadjuvant FLOT therapy and preoperatively were classified according to Clavien-Dindo (CD) grade [21]. Tumor markers, including carcinoembryonic antigen (CEA) and cancer antigen 19-9 (CA19-9), were evaluated before preoperative chemotherapy and surgery, and radiological responses to FLOT treatment were assessed with positron emission tomography and computed tomography (PET-CT). Clinical response was also assessed by pre- and postoperative endoscopic examination. Pathological response was evaluated by performing pathological examination of tissue samples taken after surgery.

### 2.2. Immunonutritional Indexes

We used mGPS as a prognostic factor, calculated based on serum albumin and CRP levels and categorized as 0, 1, and 2;

- A score of 0 for CRP serum levels within the normal range (≤10 mg/L),
- A score of 1 for high CRP serum levels (>10 mg/L) and serum albumin levels within the normal range (≥3.5 g/dL),
- A score of 2 in the presence of both high CRP serum levels (>10 mg/L) and hypoalbuminemia (<3.5 g/dL) [22].

In this study, we also calculated two additional immunonutritional indices, including PNI and geriatric nutritional risk index (GNRI), according to the following formulas:

- PNI = serum albumin (g/L) + 5 × total lymphocyte counts (10^9^/L) [23].
- GNRI = 1.487 × albumin (g/L) + 41.7 × current body weight (kg)/ideal body weight (kg). Ideal body weight = height^2^ (m^2^) × 22 kg/m^2^ [24].

### 2.3. Patient Follow-Up

Survival analysis included the time from the date of diagnosis until death caused by any reason. Data from two of the 71 included patients were lost to follow-up and could not be included in the survival analysis. The follow-up period for the mGPS 0 (*n* = 41), mGPS 1 (*n* = 14), and mGPS 2 (*n* = 14) groups was 60 months, 15 months, and 38 months, respectively.

### 2.4. Ethical Approval

Ethical approval was received from the Cukurova University Faculty of Medicine Non-Interventional Clinical Research Ethics Committee with decision number 54 on 7 April 2023.

### 2.5. Statistical Analysis

All statistical analyses were performed using Statistical Package for the Social Sciences (SPSS) software (IBM SPSS Statistics for Windows, Version 22.0. Armonk, NY, USA) and Jamovi 2.3.28 statistical software (The Jamovi project, Sydney, Australia). The normality of all variables was tested with Shapiro Wilk) test. Data are presented as mean, standard deviation, frequency, and percentage. Data were analyzed by Student’s *t*-test, Mann Whitney U test, Kruskal Wallis test, one-way analysis of variance (ANOVA), Pearson Chi-square analysis, Cox regression analysis, Kaplan Meier survival analysis, and multinomial logistic regression analysis. Statistical significance was assigned at a *p*-value of less than 0.05.

## 3. Results

We identified 71 patients with gastric cancer, aged 65 years or older, who were treated with perioperative FLOT. The mean and standard deviation for the age and BMI of the study population were 68.9 ± 4.16 years and 24.6 ± 3.82 kg/m^2^, respectively. Complete clinical follow-up for survival analysis was available for 69 patients. Patients were divided into three groups according to their pretreatment mGPS: mGPS 0 (*n* = 41), mGPS 1 (*n* = 14), and mGPS 2 (*n* = 14). The follow-up period was 60 months for the mGPS 0, 15 months for the mGPS 1, and 38 months for the mGPS 2 group. The general characteristics of patients across the mGPS are presented in Table 1. Compared with those in the mGPS 0 group, patients in the mGPS 1 and mGPS 2 groups had higher serum CRP levels (*p* < 0.001). Also, patients in the mGPS 2 group had lower albumin levels compared to mGPS 0 and mGPS 1 group (*p* < 0.001). Additionally, a significant difference was observed between the mGPS 1 and mGPS 2 groups in terms of preoperative CEA and CA 19-9 levels (*p* = 0.004). While there was no difference between mGPS 0 and mGPS 1, and mGPS 1 and mGPS 2 groups, patients with the highest mGPS values had lower PNI scores than those with the lowest (*p* < 0.001). No other significant difference was found between the mGPS groups in terms of general characteristics (*p* ≥ 0.05).

**Figure 1:**
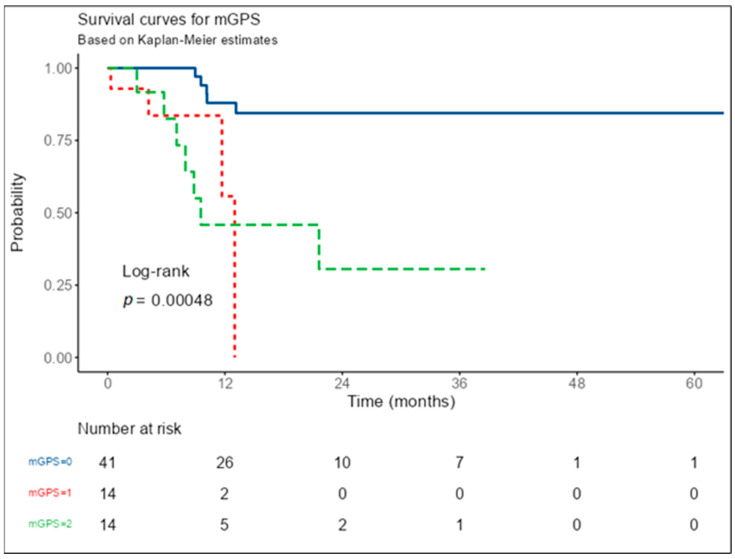
Survival analysis of patients according to mGPS group. Survival analysis was performed on 69 patients (2 patients were lost to follow-up).

As a result of the 60-month (5-year) survival follow-up of these patients, there were 5 deaths in the mGPS 0 group, four deaths in the mGPS 1 group, and seven deaths in the mGPS 2 group (Figure 1, Table 2). According to Cox regression analysis, the survival times of mGPS 1 and mGPS 2 groups were significantly shorter than mGPS 0. Compared to the mGPS 0 group, the risk of death in the mGPS 1 group was 6.25 times higher (95% CI: 1.61–24.28, *p* = 0.008) and 6.59 times higher in the mGPS 2 group (95% CI: 2.08–20.85, *p* = 0.001) (Table 2).

In the mGPS 0 group, the survival rates of the patients were 88.0% at the end of the 1st year and 84.4% at the end of the 5th year. The follow-up period of the mGPS 1 group was 15 months, and the survival rate at the end of the first year was 55.7%. While the 1-year survival rate of the mGPS 2 group was 45.8%, this rate was found to be 30.6% at the end of the 3rd year. The survival rate of the mGPS 0 group was statistically significantly higher compared to the mGPS 2 group (*p* < 0.001) (Table 3). The follow-up period was 60 months in the mGPS 0 group and 38 months in the mGPS 2 group.

As a result of the multinominal logistic regression analysis created to estimate the effect of BMI on the mGPS group, while the change in BMI did not affect the risk of being in the mGPS 1 group, it was found that each unit increase in BMI increased the risk of being in the mGPS 2 group by 1.20 times (20%) (OR: 1.20, 95% CI: 1.018 to 1.425, *p* = 0.030) (Table 4).

The relationship between mGPS and preoperative complications was examined, and there was no statistically significant difference between the mGPS groups in the elderly patients with gastric cancer treated with perioperative FLOT (*p* ≥ 0.05) (Table 5).

## 4. Discussion

In the current study, we initially explored the association between pretreatment mGPS and prognosis and overall survival in geriatric patients with gastric cancer treated with perioperative FLOT. Our results revealed that elevated pretreatment mGPS is associated with poor overall survival of gastric cancer in elderly patients treated with perioperative FLOT compared with patients with a normal mGPS. To the best of our knowledge, this is the first study focusing on the role of mGPS in predicting prognosis and survival after perioperative FLOT treatment in elderly patients with gastric cancer. We used mGPS, a two-dimensional measure of malnutrition and systemic inflammation, including positive and negative acute phase reactants (CRP and albumin), as a prognostic indicator. mGPS, which has been shown to predict poor prognosis in solid cancers, may be suitable for use in elderly cancer patients as it is also an indicator of inflammation and malnutrition associated with immunosenescence [25,26]. Recently, preoperative mGPS has been reported to be a novel and reliable predictor for overall survival and disease-free survival in surgical non-small cell lung cancer [18]. A meta-analysis of 41 clinical trials involving 18348 patients with gastric cancer found that higher mGPS was associated with poorer overall survival [27]. Similarly, the predictive effect of mGPS on overall survival has been demonstrated in different cancer types, such as pancreatic cancer [28], esophageal cancer [29], hepatocellular carcinoma [30], renal cell carcinoma [31], and lung cancer [32]. Consistent with the literature, our current study evinced that geriatric patients treated with perioperative FLOT and with elevated pretreatment mGPS experienced poorer overall survival than those with normal mGPS. A study conducted on metastatic gastric cancer patients in Turkey revealed that mGPS was superior to PNI, cachexia index, prognostic index, neutrophil-lymphocyte ratio, and sarcopenia index in predicting mortality [33]. A recent study reported that a high mGPS was a significant prognostic factor for overall survival in elderly non-small cell lung cancer patients treated with anti-programmed death 1 (PD-1) blockade (HR: 0.31 [95% CI: 0.13–0.71], *p* < 0.01) [25]. Similarly, in elderly patients with gastric cancer treated with perioperative FLOT, we showed that those with mGPS 0 had significantly longer median overall survival compared with patients with mGPS 2 (HR: 6.59 [95% CI: 2.08–20.85], *p* = 0.001).

Next, we compared the general characteristics of participants according to their mGPS group, and there was no difference in terms of age, gender, and BMI. According to tumor characteristics, there was a significant difference only in the N stages but not in the T stages. The T-stage describes the size and extent of the tumor, while the N-stage describes lymph node involvement [34]. Lymph node staging (N0/N1/N2/N3a/N3b) has been shown to be one of the most important prognostic factors in resected gastric cancer [35]. When the mGPS 0 group was compared with both the mGPS 1 and mGPS 2 groups, a significant difference emerged in the N staging of the patients. As the mGPS scores increased, the number of lymph nodes containing cancer also increased (Table 1). We compared biochemical parameters according to mGPS groups, and there were natural differences in serum CRP and albumin levels, which are part of the mGPS calculation. As expected, higher serum CRP levels and lower albumin levels were observed in the highest mGPS group. Preoperative CEA and C19-9 levels, which indicate tumor burden, were higher in the highest mGPS group than in the mGPS 0 group. High levels of these two tumor markers, which have been shown to be associated with poor prognosis in gastric cancers [36,37,38], may indicate the presence of a more aggressive tumor. 

Malnutrition is a predictor of unfavorable clinical results in older cancer patients. Several tools for screening malnutrition are available, though the optimal screening tool for this specific group remains unidentified. Based on the specific screening tool used (Nutrition Risk Screening 2002 (NRS 2002), Malnutrition Universal Screening Tool (MUST), and Mini Nutrition Assessment Short Form (MNA-SF)), the prevalence of malnutrition in older adults diagnosed with gastrointestinal cancer ranges from 20% to 52% [39]. Along with these nutritional screening tools, objective indices such as PNI, CONUT, NRI, and GNRI are used to evaluate the nutritional status of elderly cancer patients [40]. Although the prognostic importance of mGPS in cancer patients has been demonstrated in many studies, it is not among the diagnostic markers specific to malnutrition [40,41]. In our study, we showed that the PNI score was statistically significantly lower in the patients with the highest mGPS. Therefore, our findings suggested that mGPS and PNI are two tools with similar results for assessing the risk of malnutrition in elderly patients with gastric cancer receiving perioperative FLOT therapy. Another tool, the GNRI, was developed especially for the assessment of malnutrition risk in elderly patients [24]. In our study, we found that although GNRI scores tended to increase as mGPS increased, the difference was not significant.

In addition to PNI and GNRI, anthropometric markers such as weight loss and BMI are also used in the assessment of nutritional status in elderly cancer patients [40]. BMI is a simple tool that can be easily applied in clinical settings, but it does not detect the difference between fat and muscle mass and is an indicator of the total mass. Also, it should be noted that there is no consensus on any cut-off point of BMI to define obesity in the elderly [42]. In our study, the mean BMI of the mGPS 2 group was 26.4 ± 4.99 kg/m^2^. We also found that each unit increase in BMI increased the risk of falling into the mGPS 2 group by 20%. (Table 4). This relationship can be explained by the serum CRP level, one of the components of mGPS. The pathophysiological mechanism linking high BMI to elevated CRP levels involves the release of pro-inflammatory cytokines (such as IL-6) by adipose tissue, which triggers the expression and release of CRP by hepatocytes in the liver [43]. Kawamoto et al. [44] demonstrated that a BMI ≥ 25 kg/m^2^ was an independent risk factor for elevated CRP levels in adults between the ages of 65–74.

Previously, several studies have indicated that the preoperative nutritional status stands as an independent predictor of postoperative complications in geriatric cancer patients [45,46,47,48]. In a study conducted on geriatric patients with rectal cancer in Turkey, it has been reported that the mGPS and the CAR can predict serious postoperative complications and mortality [49]. Another study showed that mGPS was an independent predictor of major postoperative complications in elderly patients treated with radical cystectomy for urothelial bladder cancer [50]. A recent prospective study demonstrated that gastrointestinal cancer patients with higher mGPS were associated with postoperative complications, including requirements for blood transfusion, superficial surgical site infections, and sepsis [51]. Although the use of neoadjuvant chemotherapy in patients who underwent liver resection due to colorectal liver metastases was more prevalent in the mGPS ≥ 1 group than in those with mGPS 0, no relationship was found between preoperative mGPS and postoperative severe complications [52]. Similarly, our findings did not reveal any relationship between pretreatment mGPS and preoperative complications following neoadjuvant FLOT treatment (Table 5). No difference was observed between mGPS groups in terms of treatment-related serious side effects and chemotherapy response in geriatric patients receiving perioperative neoadjuvant FLOT treatment. In addition, similar radiological and pathological responses were observed in all three groups. In terms of toxicity, all patients’ treatments were completed, and chemotherapy was not discontinued due to serious side effects. Therefore, we speculated that perioperative neoadjuvant FLOT therapy was an effective and safe treatment regimen in geriatric gastric cancer patients. On the other hand, the limited number of cases in our study could potentially explain this result. Apart from this, our single-center retrospective study has its own limitations, such as some selection and information bias. In this study, we defined the elderly patient as ≥65 years of age, and due to our limited number of cases, we could not evaluate our results according to age ranges. On the other hand, due to the increase in life expectancy and the increase in the population of individuals over the age of 80, prospective, multicenter studies with larger samples are needed.

## 5. Conclusions

The benefit of neoadjuvant treatments in the geriatric age group is more limited due to the physiological and metabolic consequences of aging. Due to the low expectation of efficacy and high toxicity of neoadjuvant therapy in elderly patients, patients who could potentially benefit may be overlooked and deprived of the expected benefit. Nevertheless, this study evinced that high pretreatment mGPS was associated with poor overall survival in geriatric patients with gastric cancer treated with perioperative FLOT. In conclusion, pretreatment mGPS may be a simple and useful tool to predict mortality in elderly patients with gastric cancer treated with neoadjuvant FLOT. Determining prognostic factors in elderly gastric cancer patients is important not only for managing the treatment of patients but also for providing long-term benefits to patients, and further studies are required.

## Figures and Tables

**Table 1:** The characteristics of elderly patients with gastric cancer treated with perioperative FLOT (Mean ± SD or *n* [%]).

Variables	Total (*n* = 71)	mGPS 0 (*n* = 41)	mGPS 1 (*n* = 15)	mGPS 2 (*n* = 15)	*p*-Value
Age (years)	68.9 ± 4.16	69.7 ± 4.29	67.7 ± 4.18	68.0 ± 3.48	0.117
Gender (male/female)	50/21	29/12	11/4	10/5	0.982
BMI (kg/m^2^)	24.6 ± 3.82	23.7 ± 3.27	25.2 ± 3.34	26.4 ± 4.99	0.064
ECOG-PS (0/1)	60/11	33/8	13/2	14/1	0.525
Tumor subtype					
Adenocarcinoma	53 [74.6]	31 [75.6]	12 [80]	10 [66.7]	0.567
Mucinous adenocarcinoma	2 [2.8]	2 [4.9]	0	0	
Signet-ring cell carcinoma	16 [22.5]	8 [19.5]	3 [20]	5 [33.3]	
Tumor location					
Cardia	29 [40.8]	16 [39.0]	10 [66.7]	3 [20]	0.126
Corpus	14 [19.7]	8 [19.5]	1 [6.7]	5 [33.4]	
Antrum	21 [29.6]	14 [34.1]	3 [20]	4 [26.7]	
Esophagogastric junction	7 [9.9]	3 [7.3]	1 [6.7]	3 [20]	
Lauren classification (Intestinal/Diffuse)	63/8	35/6	15/0	13/2	0.250
T Stage (T1/T2/T3/T4a/T4b)	3/10/27/28/3 [4.2/14.1/38/39.5/4.2]	2/8/17/12/2 [4.9/19.5/41.4/29.3/4.9]	1/2/5/6/1 [6.7/13.3/33.3/40/6.7]	-/-/5/10/- [-/-/33.3/66.7/-]	0.374
N Stage (N0/N1/N2/N3a/N3b)	7/21/22/18/3 [9.9/29.6/31/25.3/4.2]	5/16/12/8/-[12.2/39/29.3/19.5/-]	2/3/5/3/2 [13.3/20/33.3/20/13.3]	-/2/5/7/1 [-/13.3/33.3/46.7/6.7]	0.038
Radiologic response					
Complete response	15 [21.1]	12 [29.3]	2 [13.3]	1 [6.7]	0.123
Partial response	30 [42.3]	20 [48.8]	5 [33.3]	5 [33.3]	
Stable disease	21 [29.6]	9 [22.0]	7 [46.7]	5 [33.3]	
Progressive disease	5 [7.0]	-	1 [6.7]	4 [26.7]	
Pathologic response					
Complete response	24 [33.8]	16 [39.0]	4 [26.7]	1 [6.7]	0.486
Residual disease	47 [66.2]	25 [61]	11 [73.4]	14 [93.3]	
Neutrophil count (/L)	4.97 ± 1.89	4.70 ± 1.87	4.79 ± 1.78	4.97 ± 1.67	0.996
Lymphocyte count (/L)	1.94 ± 1.15	1.71 ± 0.49	1.79 ± 0.46	1.97 ± 0.95	0.807
Hemoglobin (g/dL)	12.1 ± 2.03	11.7 ± 1.87	12.1 ± 2.87	12.2 ± 1.52	0.730
CRP (mg/dL)	10.0 (0.34–156.0)	4.5 (2.0–10.0) ^a^	15.0 (12.0–30.0) ^b^	31.0 (16.0–65.0) ^b^	˂0.001
Albumin (g/dL)	3.59 ± 0.47	3.68 ± 0.39 ^a^	3.83 ± 0.18 ^a^	2.97 ± 0.31 ^b^	˂0.001
Preoperative CEA (ng/mL)	4.0 (0.33–74.0)	3.49 (0.51–9.30) ^a^	2.85 (0.33–74.0) ^a,b^	6.1 (1.59–42.0) ^b^	0.027
Preoperative CA 19-9 (U/mL)	11 (0.3–2307.0)	5.20 (0.3–276.0) ^a^	13.5 (0.4–2307.0) ^a,b^	60.0 (0.5–1792.0) ^b^	0.004
PNI	36.2 ± 4.78	37.3 ± 3.99 ^a^	38.7 ± 2.87 ^a,b^	30.5 ± 4.07 ^b^	˂0.001
GNRI	52.0 ± 7.23	50.5 ± 6.32	53.5 ± 6.42	54.7 ± 9.47	0.139

Values with different superscripts (^a, b^) in the same row indicate significant differences as a result of post hoc analysis. mGPS, modified Glasgow prognostic score; SD, standard deviation; BMI, body mass index; ECOG-PS, Eastern Cooperative Oncology Group (ECOG) Performance Status; CRP, C-reactive protein; CEA, carcinoembryonic antigen; CA 19-9, cancer antigen 19-9; PNI, prognostic nutritional index; GNRI, geriatric nutritional risk index.

**Table 2:** Median Survival Table: Levels for mGPS.

	Numbers of Event		Time (Months)	Cox Table-mGPS	Pairwise Comparisons mGPS	
Levels	Records	Events	Median	HR (95% CI)	Level	*p*-Value
mGPS 0	41	5	NA	-	1-0	0.005
mGPS 1	14	4	13.00	6.25 (1.61–24.28), *p* = 0.008	2-0	<0.001
mGPS 2	14	7	9.53	6.59 (2.08–20.85), *p* = 0.001	2-1	1.000

*p*-value adjustment method: Bonferroni. mGPS, modified Glasgow prognostic score; HR, hazard ratio; CI, confidence interval. Survival analysis was performed on 69 patients (2 patients were lost to follow-up).

**Table 3:** 1-year, 3-year, and 5-year survival rates of patients according to mGPS group.

Levels	Time (Months)	Number at Risk	Number of Events	Survival	Lower	Upper
**mGPS 0**	12	26	4	88.0%	77.6%	99.8%
	24	10	1	84.4%	72.7%	98.0%
	36	7	0	84.4%	72.7%	98.0%
	48	1	0	84.4%	72.7%	98.0%
	60	1	0	84.4%	72.7%	98.0%
**mGPS 1**	12	2	3	55.7%	24.1%	100.0%
**mGPS 2**	12	5	6	45.8%	24.1%	87.2%
	24	2	1	30.6%	10.9%	85.3%
	36	1	0	30.6%	10.9%	85.3%

mGPS, modified Glasgow prognostic score.

**Table 4:** Multinomial logistic regression analysis of the association between BMI and mGPS.

mGPS	Predictor	Estimate	SE	Z Score	*p*-Value	OR	95% Cl
1-0	Intercept	−3.803	2.1284	−1.79	0.074	0.02229	3.44–1.445
	BMI	0.115	0.0854	1.34	0.179	1.12149	0.949–1.326
2-0	Intercept	−5.706	2.1962	−2.60	0.009	0.00333	4.49–0.246
	BMI	0.186	0.0859	2.16	**0.030**	1.20426	1.018–1.425

mGPS, modified Glasgow prognostic score; BMI, body mass index; SE, standard error; OR, odds ratio; CI, confidence interval.

**Table 5:** The relationship between mGPS and preoperative complications in elderly patients with gastric cancer treated with perioperative FLOT.

	mGPS 0 (*n* = 41)			mGPS 1 (*n* = 15)			mGPS 2 (*n* = 15)			
	None	G1–2	G3–4	None	G1–2	G3–4	None	G1–2	G3–4	*p*-Value
Dose deferral	29 [70.7]	12 [29.3]	-	8 [53.3]	7 [46.7]	-	12 [80.0]	3 [20.0]	-	0.162
G-CSF prophylaxis	6 [14.6]	35 [85.4]	-	2 [13.3]	13 [86.7]	-	-	15 [100.0]	-	0.320
Grade 3/4 toxicity	30 [73.2]	11 [26.8]	-	11 [73.3]	4 [26.7]	-	13 [86.7]	2 [13.3]	-	0.294
Dose reduction	26 [63.4]	15 [36.6]	-	10 [66.7]	5 [33.7]	-	12 [80.0]	3 [20.0]	-	0.295
Fatigue	9 [22]	32 [78.0]	-	5 [33.3]	9 [60.0]	1 [6.7]	1 [6.7]	14 [93.3]	-	0.135
Hand-foot syndrome	29 [70.7]	12 [29.3]	-	9 [60.0]	6 [40.0]	-	13 [86.7]	2 [13.3]	-	0.306
Neutropenia	23 [56.1]	14 [34.1]	4 [9.8]	7 [46.7]	4 [26.7]	4 [26.7]	11 [73.3]	4 [26.7]	-	0.150
Anemia	19 [46.3]	21 [51.2]	1 [2.4]	8 [53.3]	7 [46. 7]	-	4 [26.7]	10 [66.6]	1 [6.7]	0.593
Thrombocytopenia	28 [68.3]	13 [31.7]	-	12 [80.0]	3 [20.0]	-	9 [60.0]	6 [40.0]	-	0.610
Febrile neutropenia	33 [80.5]	8 [19.5]	-	11 [73.3]	4 [26.7]	-	12 [80.0]	3 [20.0]	-	0.702
Mucositis	23 [56.1]	17 [41.5]	1 [2.4]	8 [53.3]	7 [46.7]	-	12 [80.0]	2 [13.3]	1 [6.7]	0.293
Diarrhea	13 [31.7]	27 [65.9]	1 [2.4]	4 [26.7]	11 [73.3]	-	3 [20.0]	11 [73.3]	1 [6.7]	0.846
Neuropathy	21 [51.2]	19 [46.3]	1 [2.4]	8 [53.3]	7 [46.7]	-	8 [53.3]	7 [46.7]	-	0.937
Nausea	7 [17.1]	33 [80.5]	1 [2.4]	3 [20.0]	12 [80.8]	-	1 [6.7]	13 [86.6]	1 [6.7]	0.773
Vomiting	15 [36.6]	25 [61.0]	1 [2.4]	6 [40.0]	9 [60.0]	-	7 [50.0]	7 [50.0]	1 [6.7]	0.845
Stomatitis	29 [70.7]	11 [26.8]	1 [2.4]	12 [80.0]	3 [20.0]	-	11 [73.3]	4 [26.7]	-	0.887
Allergic complications	36 [87.8]	5 [12.2]	-	12 [80.0]	3 [20.0]	-	14 [93.3]	1 [6.7]	-	0.223
Thrombosis	36 [87.7]	5 [12.2]	-	14 [93.3]	1 [6.7]	-	15 [100.0]	-	-	0.355
Renal toxicity	36 [87.8]	5 [12.2]	-	15 [100.0]	-	-	14 [93.3]	1 [6.7]	-	0.767
Hepatic toxicity	36 [87.8]	5 [12.2]	-	15 [100.0]	-	-	14 [93.3]	1 [6.7]	-	0.355

mGPS, modified Glasgow prognostic score; G, grade; G-CSF, granulocyte-colony stimulating factor.

## Data Availability

Data are available on request from the authors.
